# Use of the sit-to-stand task to evaluate motor function of older adults using telemetry

**DOI:** 10.1186/s12877-016-0294-2

**Published:** 2016-06-06

**Authors:** Akira Kanai, Sachiko Kiyama, Hiroshi Goto, Hidehito Tomita, Ayuko Tanaka, Mitsunobu Kunimi, Tsutomu Okada, Toshiharu Nakai

**Affiliations:** Graduate School of Health Sciences, Toyohashi SOZO University, 20-1 Matsusita, Ushikawa-cho, Toyohashi, 440-8511 Japan; Department of Physical Therapy, School of Health Sciences, Toyohashi SOZO University, 20-1 Matsusita, Ushikawa-cho, Toyohashi, 440-8511 Japan; NeuroImaging & Informatics, National Center for Geriatrics & Gerontology, 7-430 Morioka-cho, Ohbu, 474-8511 Japan; Aichi Prefectural Hospital and Rehabilitation Center for Disabled Children, Dai-ni Aoitorigakuen, 5-1 Aza-yanagisawa, Okazaki, 444-3505 Japan; School of Humanities, College of Human and Social Science, Kanazawa University, Kakuma-machi, Kanazawa, 920-1192 Japan

**Keywords:** Older adults, Sit-stand-task, Physical batteries, Iliac elevation maximum velocity, Knee extension maximum angular velocity, Multi-group structural equation modeling, Remote motion capture

## Abstract

**Background:**

Physical exercises are widely used in community programs, but not all older adults are willing to participate. Information and communication technology may solve this problem by allowing older people to participate in fitness programs at home. Use of remote instruction will facilitate physical exercise classes without requiring that participants gather at one place. The aim of this study was to examine use of a sit-to-stand task in evaluating motor function using conventional video communication in a telemetry system to enable real-time monitoring, and evaluation in physical performance of older adults at home.

**Methods:**

The participants were 59 older individuals and 81 university students. Three physical exercise batteries were used: arm curl, figure-of-eight walk test, and functional reach. The knee extension maximum angular velocity (KEMAV) and the iliac elevation maximum velocity (IEMV) during standing up from a chair and the heel rise frequency were used in the motion-capture measurements. The results were assessed using multi-group structural equation modeling (SEM) for the young and older groups.

**Results:**

Young participants consistently performed better than their older counterparts on all items. Analyses with multi-group SEM based on correlations between items yielded a good model-fit for the data. Among all path diagrams for IEMV and KEMAV in the older and young groups, paths from muscular strength to skillfulness showed significant effects. The path from the IEMV to muscular strength was also significant in the older group.

**Conclusions:**

Multi-group SEM suggested that video-based measurements of IEMV during sit-to-stand motion can estimate muscular strength, which suggests that remote monitoring of physical performance can support wellness of community-dwelling older adults.

## Background

Physical therapy in geriatrics has an important role as a prophylactic intervention using various physical exercises to maintain and improve motor function. In particular, recovery of physical activity related to daily life is important for older adults because a high activity level is related to a high physical function [24]. Fall prevention is also an important purpose of physical exercise intervention [2, 9, 23, 38]. A review of 17 studies concluded that exercise programs designed to prevent falls in older adults prevent injuries caused by falls and reduce the rate of falls requiring medical care [2].

Physical exercise courses are widely used in community programs, but problems include the effort required for continuation of learned exercises at home, and the lack of willingness of some older adults to participate in a course. Improvement in gait ability was observed after low-fitness older adults underwent 6-month low-strength muscular training such as chair exercise in a group; however, the effects did not persist for more than 3 months after direct instruction was stopped, suggesting that the participants did physical exercises during the program period, but may not have continued these exercises at home [19]. Therefore, it would be beneficial to develop a system to monitor the physical status of older people after a course to maintain connection to the program.

One solution for the problems above is to introduce information and communication technology (ICT) that enables older people to participate in fitness programs at home. Rapid developments in processing power, data transmission, and data storage in interactive technologies have led to a continual evolution of eHealth interventions [26]. Physical exercises and nutrition programs using communication technologies have been designed using eHealth platforms. For example, the low cost and greater accessibility of computer-based telecommunication exercise interventions led to improvements in strength, balance, and depressive symptoms [40], and instruction in an online aftercare program for cardiac rehabilitation increased the physical activity of older patients [30]. These studies show that online programs can improve cardiac rehabilitation and indicate that physical exercise ICT programs can support self-management of various health disorders.

ICT programs allow instructions on physical exercise to be given without requiring the participants to gather at one place, and minimize or eliminate the cost and time of instructors visiting community-dwelling older adults door-to-door. However, valid remote monitoring of movement requires sufficient time, spatial resolution and quantitation. Therefore, the motions for such remote measurements should be as simple, reproducible, and safe as possible for older people to perform. In this study, we designed a telemetry approach to evaluate the relationship between performance of sit-to-stand movement, which mostly reflects the strength of the knee and hip joint, and physical activity of older subjects [3, 16, 22, 47, 48]. Yoshida et al. [46] compared the strength of the knee extension muscles and maximal gait speed in participants and non-participants in a physical exercise program, and found lower performance in male and female non-participants. Sit-to-stand movement is common in daily life and performance of this motion for measurement does not increase risk of accidents.

As a battery for physical activity, we used the protocol used by Shigematsu et al. [37], in which it was proposed that physical status that reflects daily life activity can be predicted using functional fitness tests such as the arm curl (AC), figure-of-eight walk (F8W) test, and functional reach (FR). The time and number of steps in the F8W test is significantly correlated with the time in the 10-m walking test and the Timed Up and Go test [37]. F8W is also correlated with other walking parameters in older adults, such as gait speed, physical function, confidence in walking and movement control [18]. However, the F8W test is unsuitable for a telemetry system because of the risk of falls, complexity of movement, and the space required for the test. From this point of view, the sit-to-stand task test may be a useful tool to assess balance and the risk of falls in older people [5]. Concentric strength at higher velocity, eccentric strength, and rate of torque development are important contributors in sit-to-stand performance [7]. The rate of body rise is hypothetically a useful index of exercise capacity for older adults, and thus, the sit-to-stand task is appropriate for telemetry systems.

To evaluate the rate of rise by telemetry, we focused on two markers: (1) the knee extension maximum angular velocity (KEMAV) as a marker of body rise, and (2) the iliac elevation maximum velocity (IEMV) to reflect the gravity center of the body. In addition, we measured the frequency of heel rise (HR) to reflect the strength of the lower limb muscles [17, 34]. The HR task can also be easily performed without relocating the position, similarly to the sit-to-stand task. Thus, the sit-to-stand and HR tasks can be performed within the monitoring range of a conventional webcam. Most physical exercises for fall prevention use gait, balance and functional training to recover physical activity of the lower extremities [2, 9]. By comparing the performance on a sit-to-stand task by video-monitoring and the actual physical activity of the subjects, the potential of telemetry for home monitoring using a conventional video communication device was investigated.

## Methods

### Participants

The participants were 59 older adults (26 males, 33 females; mean age 74 ± 3.3 years) and 81 university students (44 males, 37 females; mean age 20.8 ± 0.9 years). The older participants were recruited in a physical exercise classroom at a local community club. All participants were neurologically normal and had no physical disabilities. Written informed consent was obtained from all participants in accordance with the research protocol approved by the institutional review board of the National Center for Geriatrics and Gerontology (Protocol #495, Dec 2010) in accordance with the ethical standards laid down in the Declaration of Helsinki.

### Physical test batteries and movement tasks

The AC, F8W, and FR tasks were used as the physical batteries set. These tests were performed twice and the better value of the two measurements was used to represent the maximal effort, following the protocol used by Shigematsu et al. [37]. Two motion-capture recordings were made to simulate remote measurements. The first motion recorded was standing up from a chair to measure KEMAV and IEMV. To start this motion, the height of the chair bench was adjusted manually based on the length of the individual’s lower extremity. The subject folded arms in front of the chest and was instructed to quickly start the stand up motion in response to a flashing light signal of 1 s duration that was placed 0.8 m above and 1 m in front of the chair. The second motion recorded was the HR to evaluate the strength of the lower limb muscles. The participants performed self-paced HR as fast as they could for 10 s in a standing position, and the number of repetitions was recorded. Arms were crossed on the chest during the HR task to standardize the center of gravity.

### Motion-capture measurements and image analysis

**Fig. 1:**
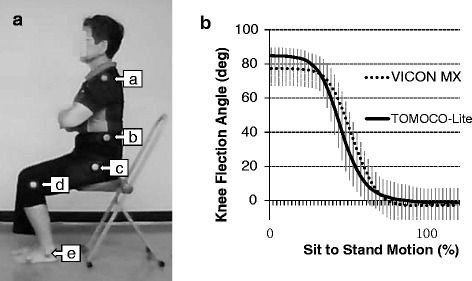
**a**. Video recording of sit-to-stand motion. The motion was acquired from the left of the body, and the iliac elevation maximum velocity (IEMV) and knee extension maximum angle velocity (KEMAV) were measured using five color markers placed at the acromion (*a*; *red*), iliac crest (*b*; *green*), greater trochanter (*c*; *yellow*), lateral epicondyle of the femur (*d*; *red*), and lateral malleolus (*e*; green). The height of the chair is adjusted according to the length of the lower extremity. **b**. Comparison of webcam and VICON MX measurements of knee angle change during sit-to-stand motion. The solid line shows the average of 42 measurements using a webcam and the dashed line shows that with VICON MX. The temporal change of the knee angle was interpolated using 51 points (2 % for each step) to cover the whole sit-to-stand motion. The correlation coefficient (r) between the time series was 0.920 ± 0.089 (*n* = 42). Error bar: standard deviation

Color markers for motion tracking were attached to the left side of the acromion (red); iliac crest (green); greater trochanter (yellow); lateral epicondyle of the femur (red); and lateral malleolus (green) (Fig. 1). Motion was recorded using a network CCD webcam (CG-WLNCPTGL; Corega, Yokohama, Japan) and standard video-recording software (NC Monitor; Corega). The webcam was directly connected to a laptop PC to record via an ethernet port (100 Mbps). Motions were recorded at a resolution of 480 (vertical) × 640 (horizontal) pixels (30 frames/s, angle of view: 50° in horizontal and 37° in vertical directions, focal length = 4.0 mm, *F* = 1.8, compression: motion Joint Photographic Experts Group (JPEG), recorded in Audio Video Interleave (AVI) format). The webcam was located 80 cm above the floor with its optical axis directed horizontally to monitor the left side of the participant at a distance of 5 m. The length per pixel was calibrated by putting a rod (1 m) at the position the subjects were sitting.

To extract the trajectories of the color markers, video-recordings were analyzed using two-dimensional (2D) motion analysis software (Tomoko-Lite; Toso System, Saitama, Japan) [28]. On the first frame, an experienced operator manually assigned each color marker using manual-tracking mode to extract the gravity centers of the markers in the 2D coordinates. Then, the markers were automatically tracked from the second frame. To obtain IEMV (cm/s), the marker located at the center of iliac crest was selected and its maximum vertical velocity was calculated. To compute KEMAV, markers at the greater trochanter, lateral epicondyle and lateral malleolus were selected and the maximum angular velocities (degree/s) between the greater trochanter-lateral epicondyle and lateral epicondyle-lateral malleolus were computed.

A benchmark test was performed to verify the reliability of measurement using the webcam. The knee angle change during sit-to-stand motion was compared between this system and VICON MX (Vicon Motion Systems, Oxford, UK). Fourteen young subjects (7 males, 7 females; age 20.4 ± 0.5 years) repeated sit-to-stand motion 3 times and the change in temporal knee angle change was simultaneously recorded by the two systems.

### Data analyses with multi-group structural equation modeling

The purpose of the analyses was to compare the applicability of video-recorded IEMV and KEMAV indices as candidate predictors of intrinsic physical abilities of older adults. To achieve this, we first assumed two sets of path diagrams in which IEMV or KEMAV directly predicts muscular strength, which further predicts physical skillfulness. The validity of this assumption was assessed by multi-group structural equation modeling (SEM) in the young and older groups. SEM is a technique used to estimate causal relationships among multiple variables, based on their covariance structures (Schumacker and Lomax [35]). In contrast to simply repeating statistical tests of regressions or correlations, the advantage of SEM is that it simultaneously evaluates the goodness-of-fit between the assumed model and the given data. Even if some regression weights are significant, the assumed model is not statistically acceptable unless the goodness-of-fit indices (GFIs) meet the conventional criteria, which support the robustness of the model as a whole. In addition, SEM permits multi-group analyses by direct comparisons of estimated parameters across groups [1]. AMOS 21 J software (SPSS, Chicago, IL, USA) was used to analyze the data using the maximum likelihood estimation method.

### Procedure for multi-group SEM

#### Model assumption

Three variables were used to postulate each path diagram: skillfulness, muscular strength, and IEMV or KEMAV. SEM can take into account observed and latent (i.e. unobserved) variables. The first two variables were considered to be latent since they are inferred from an observed variable or variables; Thus, one set of observed variables, WF8 and FR, was used to infer skillfulness, and another set, AC and HR, was used to infer muscular strength. The third variable was used to determine whether IEMV or KEMAV is more predictive of the physical abilities of older adults. For this, each of the two indices was alternately entered into the path diagrams as an observed variable with the other variables remaining constant.

#### Cross-group constraint for multi-group analysis

Performing multi-group SEM requires anticipating whether each path is equivalent across groups. If a path is anticipated to be equivalent, a cross-group constraint has to be imposed on the path. For the many possible cross-group constraints among the paths, four models were tested to find the one with the best GFI. Model 1 assumed a measurement equivalence in which cross-group constraints were imposed on the paths between each latent variable and its constituent observed variables (i.e., the paths from WF8/FR to skillfulness and from AC/HR to muscular strength), whereas other paths were not constrained. Model 2 added a constraint on the path between the two latent variables (i.e., muscular strength and skillfulness) in Model 1, whereas the path from IEMV/KEMAV to muscular strength was not constrained. Model 3 added a path from IEMV/KEMAV to muscular strength, instead of the path between the latent variables constrained in Model 2. Model 4 imposed constraints on all parameters.

#### Model comparison

Comparisons were performed to find the best model for each diagram. Among the four candidate models, the Akaike information criterion (AIC) and Browne-Cudeck criterion (BCC) were evaluated because these criteria are indices for direct model comparisons. Their relative differences, rather than absolute values, indicate a better fit (the smaller, the better). In addition, the fit of the model to the data was tested using the chi-square (*χ*^*2*^) statistic, in which insignificance supports homogeneity (i.e. supports fit). In addition to the chi-square test, the goodness-of-fit is evaluated by other indices, such as the GFI and the comparative fit index (CFI), which are conventionally required to exceed 0.95. The root mean square error of approximation (RMSEA) should also be <0.1 (Schumacker and Lomax [35]).

#### Inspection of the best model

Once we obtained the models that gave the best solution for each of the path diagrams (i.e., IEMV and KEMAV) for the young and older groups, we looked for significant pass coefficients. A path that had no cross-group constraint indicated that the path value differed across groups. For such paths, pairwise parameter comparisons based on *z*-scores were conducted to examine whether the path values differed significantly between the groups [1].

## Results

A comparison of video-based measurements between the webcam and VICON MX is shown in Fig. 1b. The temporal change of the knee angle was interpolated using 51 points (2 % for each step) to cover the whole sit-to-stand motion procedure. A series of ANOVAs with repeated measures (the type of camera repetition of measurement) were applied for each of the 51 points. The angle of the knee joint depended on the type of camera from the first to the 30th point. However, no significant difference was found after the 31st point. No significant effect of repetition of measurement or interaction between the two factors was throughout all 51 points. The correlation coefficient (r) between the two time series was 0.920 ± 0.089 (*n* = 42).

All young and older participants performed the AC, FR and F8W physical batteries and sit-to-stand and HR tasks appropriately. IEMV and KEMAV were obtained for all participants by analyzing the video-recorded data. Scores on the physical examinations for the young and older groups (Table 1) indicated that young participants consistently performed better in comparison to their older counterparts (paired *t*-test). The results of the physical performance tests in the older group were consistent with results reported by Shigematsu et al. [36].

**Table 1:** Physical characteristics of the participants and results of physical performance

	Young (*n* = 81)	Elderly (*n* = 59)	*t*-value	*p*-value
Age (years)	20.8 ± 0.9	74.1 ± 5.9	−79.4	*p* < 0.001
Height (cm)	165.9A ± 8.9	155.5 ± 8.4	6.8	*p* < 0.001
Weight (kg)	58.9 ± 10.4	54.8 ± 10.0	2.3	0.024
BMI (kg/m^2^)	21.3 ± 3.0	22.6 ± 3.4	−1.78	0.080
F8W test (sec)	18.0 ± 2.4	26.9 ± 5.1	−13.9	*p* < 0.001
Functional reach (cm)	35.7 ± 5.4	30.6 ± 5.2	5.7	*p* < 0.001
Arm curl (times)	32.6 ± 6.3	24.2 ± 4.8	8.6	*p* < 0.001
Heel rise test (times)	26.6 ± 6.1	24.3 ± 7.1	2.1	0.041
Illiac elevation maxmum velocity (cm/s)	151.1 ± 27.9	116.4 ± 22.8	7.8	*p* < 0.001
Knee extension maximal angular velocity (deg/s)	279.6 ± 52.3	239.3 ± 50.1	4.6	*p* < 0.001

The performance of young adults was significantly better than that of older adults for F8W, FR, AC, IEMV and KEMAV (*p* < 0.001). Values are shown as the mean ± standard deviation. The two age groups were compared using t-statistics. df = 138

Analyses with multi-group SEM based on correlations between items (Table 2) yielded models with a good fit to the data (Table 3). For the path diagram with IEMV, Model 1 indicated the best fit, based on having the lowest AIC and BCC among the four candidate models. The strength of causal relationships among the three variables differed across the older and young groups because Model 1 imposed no cross-group constraints among IEMV, muscular strength, and skillfulness. On the other hand, Model 3 gave the best solution in the diagrams with KEMAV; this model imposed a cross-group constraint on the path from KEMAV to muscular strength, but not on the path from muscular strength to skillfulness. This finding indicates that the path from KEMAV to muscular strength is equivalent across the two groups, whereas the path from muscular strength to skillfulness is not.

**Table 2:** Correlations among the 6 behavioral datasets in each age group

A) Pearson coefficient of correlation (r) between the measurement items in elderly people (*n* = 59)						
	F8W^a^	FR^b^	AC^c^	HR^d^	IEMV^e^	KEMAV^f^
F8W	-					
FR	-.524^***^	-				
AC	-.358^**^	.375^**^	-			
HRT	-.505^**^	.459^***^	.521^***^	-		
IEMV	-.310^*^	.331^*^	.310^*^	.391^**^	-	
KEMAV	-.300^*^	.265^*^	.047	.176	.706^***^	-
B) Pearson coefficient of correlation (r) between the measurement items in young people (*n* = 81)						
	F8W	FR	AC	HRT	IEMV	KEMAV
F8W	-					
FR	-.340^**^	-				
AC	-.538^***^	.318^**^	-			
HRT	-.460^***^	.320^**^	.523^***^	-		
IEMV	-.051	.321^**^	.268^*^	.165	-	
KEMAV	-.099	.167	.107	.064	.453^***^	-

Correlations among the behavioral datasets were obtained in multi-group SEM analysis for each age group. *p < .05, **p < .01, ***p < .001, a: Figure-of-eight walk test, b: Functional reach, c: Arm curl, d: Heel rise, e: Iliac elevation maximum velocity, f: Knee extension maximal angular velocity

**Table 3:** Fit indices for model comparisons

Model assumption	AIC	BCC
Models concerning *IEMV*		
Model 1 (measurement equivalence)	57.917	61.865
Model 2 (constrained between *muscle strength* and *skillfulness* in addition to Model 1)	58.903	62.635
Model 3 (constrained between *IEMV* and *muscle strength* in addition to Model 1)	59.373	62.925
Model 4 (fully constrained)	59.373	62.925
Models concerning *KEMAV*		
Model 1 (measurement equivalence)	53.212	57.160
Model 2 (constrained between *muscle strength* and *skillfulness* in addition to Model 1)	54.465	58.214
Model 3 (constrained between *KEMAV* and *muscle strength* in addition to Model 1)	51.541	55.291
Model 4 (fully constrained)	52.590	56.143

The extent of fit was compared among four SEM models with different constraints for the two movement parameters obtained using a webcam. The lower value of the fit index represents better fit of the model. AIC: Akaike information criterion; BCC: Browne-Cudeck Criterion. IEMV: iliac elevation maximum velocity; KEMAV: knee extension maximum angular velocity

**Fig. 2:**
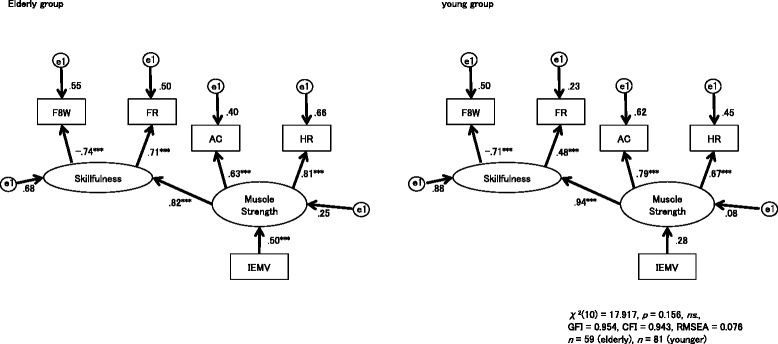
Path diagrams for the iliac elevation maximum velocity (IEMV) in the older and young groups. Paths from muscular strength to skillfulness showed a significant effect in each group (*p* < 0.001). The path from IEMV to muscular strength was significant in the older group (*β* = 0.50, *p* < 0.001), but not in the young group (*β* = 0.28). GFI: goodness-of-fit index, CFI: confirmatory fit index, RMSEA: root mean square error of approximation

**Fig. 3:**
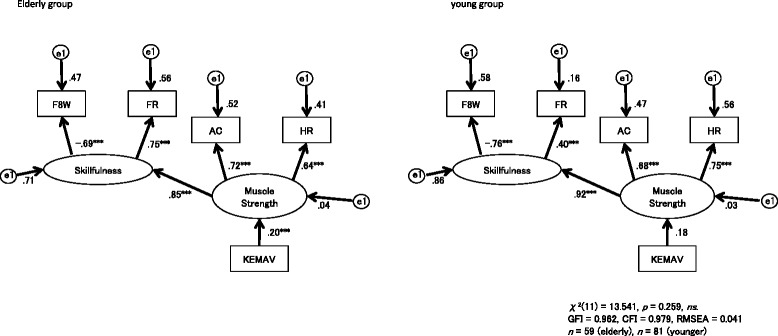
Path diagrams for the knee extension maximum angle velocity (KEMAV) in the older and young groups. Paths from muscular strength to skillfulness showed a significant effect in each group (*p* < 0.001). The path from the iliac elevation maximum velocity (IEMV) to muscular strength was significant in the older group (*β* = 0.20, *p* < 0.001), but not in the young group (*β* = 0.18). GFI: goodness-of-fit index, CFI: confirmatory fit index, RMSEA: root mean square error of approximation

The best models for IEMV and KEMAV are shown in Figs. 2 and 3, respectively. Both models had adequate GFIs (IEMV, *χ*^*2*^_10_ = 17.917, *p* = 0.156, *ns*, GFI = 0.954, CFI = 0.943, RMSEA = 0.076; KEMAV, *χ*^*2*^_11_ = 13.541, *p* = 0.259, *ns*, GFI = 0.962, CFI = 0.979, RMSEA = 0.041). For all IEMV and KEMAV diagrams in the older and young groups, the paths from muscular strength to skillfulness had significant effects (*p* < 0.001). The path from IEMV to muscular strength in the older group was also significant (*β* = 0.51, *p* < 0.001), but pairwise parameter comparison of this path between the two groups did not give a significant *z*-score.

## Discussion

Fall prevention is an important aim of group exercises as a health promotion program in local communities [2, 8, 9, 29]. For example, 12 weeks of incremental training of the lower limb was shown to improve fall-related gait kinematic parameters [29]. Having participants get together in one place for group exercises, checking their physical activities, and giving them instructions is time-effective. However, some community-dwelling older adults are unwilling to join such programs [31]. Remote instruction is likely to be useful for such people and for following up with older patients after a physical exercise program [30]. However, observation of physical activity using eHealth technologies has several limitations, such as restriction in the spatial data dimension, risk management to prevent falls during the session, and standardizing regulation of motor performance. Therefore, in this study, we designed a model for remote physical examination using the sit-to-stand task, which is an easy and safe movement controlled by simple instructions.

The sit-to-stand task has been used to evaluate knee muscular strength, which is associated with fall risk [3, 5, 7]. The physical dynamics and correlation between knee muscle strength and sit-to-stand motion has been investigated using devices such as a leg press and knee extensors [3], flywheel [16], dynamometer [7, 21, 46], force plate [5, 20, 25, 47] and weightlifting analyzer [14]. The peak floor reaction force in sit-to-stand motion occurs during knee extension movement soon after seat clearance [25]. The better the ability for the sit-to-stand task, the greater the muscle strength for pushing against the floor. Sit-to-stand velocity is related to lower limb muscular strength [3, 18, 22, 47, 48] and this tendency is clear in comparison of sit-to-walk velocity between older and young individuals Kouta and Shinkoda [20] Application of a weightlifting analyzer is a unique method Gray and Paulson [14] that combined a force plate and cinematography to calculate the change of center of mass during sit-to-stand motion more accurately, since it takes into account deficits in balance that cause an older adult to rise more slowly when performing a seated power movement. These methods showed clear relationships among indices obtained from sit-to-stand motions, but used three-dimensional (3D) motion capture systems with multiple high-resolution cameras and physical devices for muscular strength measurement. In contrast, we investigated the utility of a conventional network camera to monitor one or a few sit-to-stand motions at home or in a community meeting place.

We used three indices for physical activity of older adults (AC, FR, F8W) [36, 37] and one for lower limb muscle strength (HR), all of which can be obtained without special measurement devices, such as a dynamometer or force plate. AC and HR have been suggested to reflect muscular strength, while F8W and FR reflect skillfulness in motion [17, 18, 34, 36, 37]. The advantage of these four indices is that muscular power and skillfulness, the two basic elements required to generate movement, can be used as latent variables in the SEM model. Using the four observable variables, the performances of IEMV and KEMAV, two image-based indices of sit-to-stand motion, were compared as predictors of muscular power and skillfulness. The SEM analysis suggested that IEMV better predicts muscular power (Fig. 2) than KEMAV (Fig. 3) in older adults and that muscular power predicts skillfulness in both age groups. Skillfulness of motion manifests as balance, motor coordination, flexibility and speed, and has a strong association with cognitive function [44]. Since balance is the key factor in fall risk evaluation Rubenstein [32], an evaluation model including both muscular power and skillfulness will be useful for future investigation of fall prevention in older adults with potential cognitive decline.

**Fig. 4:**
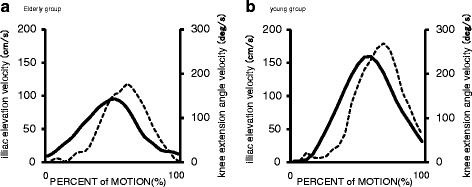
Curves illustrating the iliac elevation velocity (IEMV, solid line) and knee extension angular velocity (KEMAV, broken line). **a** Young group. **b** Older group. The timing of KEMAV during sit-to-stand motion was delayed compared to IEMV in both groups

IEMV and KEMAV were utilized independently, rather than in one model, for two reasons. First, it would be useful to use just the better index for screening. Second, since IEMV and KEMAV both reflect the result of the same knee joint extension, but in a geometrically different view, it is reasonable to choose the better index rather than using two replicated indices. KEMAV during sit-to-stand movement did not significantly predict muscular strength, although it reflects the same movement as IEMV. This discrepancy may be due to the difference in the temporal characteristics of the two indices in response to the movement. KEMAV directly depends on angular movement by knee muscle constriction in the 2D plane, whereas IEMV is a consequence of movement only in the perpendicular (cosine) projection at the distal end point of the angular movement (Fig. 1). Therefore, the temporal characteristics of the two velocity parameters can manifest differently depending on the phase of sit-to-stand motion (Fig. 4).

We included HR as a manual muscle test of the lower extremity, since the HR test is commonly used to evaluate the strength of the triceps surae muscles. HR may also be a useful index for evaluating exercise capacity due to its strong correlation with other physical battery tests (Table 2) and its reflection of muscular strength, as for AC (Figs. 2 and 3). However, HR is an unstable motion with a potential risk of fall when performed by older adults without assistance. Therefore, sit-to-stand motion is preferable to HR for remote monitoring at home.

Another advantage of sit-to-stand motion is that it may reflect the general status of older adults, other than fall risk. In a study of nursing home residents with dementia, higher scores on the 30-s sit-to-stand task and the Berg Balance Scale were associated with a better quality of life (QOL) on the late-stage dementia scale [42]. It was also suggested that the chair stand test may be as useful as the complete Short Physical Performance Battery in estimating mortality risk [4]. In addition to evaluating the physical status of older adults, sit-to-stand motion can be used as a physical intervention. As a daily care routine, sit-to-stand activity in a nursing home maintained mobility and function and slowed functional decline of residents with dementia [39]. These studies suggest that analysis of sit-to-stand motion may provide information related to activities of daily living.

This study showed that quantification of sit-to-stand movement using a webcam can estimate muscular power and skillfulness of older adults. However, 2D measurement using one camera had some discrepancy with that by 3D measurement (Fig. 1b), and several improvements should be considered for practical use. One limitation of the study was that all preparations and measurements were performed by experimenters and the study design did not include potential confounders that depend on an individual, such as the precision of the marker and camera positions, the height of the seat, and the network environment to collect the video images. To remove these potential errors in remote monitoring, it will be necessary to establish a protocol to calibrate measurements within a reasonable time. Older adults will need support for initial setup of the measurement by trained personnel, including choice of webcam location, spatial calibration, and instructions for attaching markers and performing sit-to-stand movement. We set up the webcam 5 m from the subject to cover the whole body, but the minimum space for measurement can be smaller. This distance can be shortened by setting the webcam in vertical alignment and limiting the field of view to cover just the markers. By fixing the webcam at a constant location and setting markings on the floor, measurements will be reproducible. It may not be easy to attach markers at proper positions for older adults, but only a few markers are required to measure sit-to-stand motion. On-line instruction to adjust the marker positions will be a primary need for some people and visits by trained personnel may be needed.

Markerless motion capture [12, 13] with a 3D body model (Samantha et al. [33]) may be an alternative approach, since subjects do not have to locate several markers precisely. There have been many attempts to apply Kinect, a motion sensing input device that provides a user interface using gesture and spoken commands, to physical evaluation such as gait assessment [11, 41], Timed Up and Go [43], exergames for fall prevention at home [15], and balance tests for stroke patients Clark et al. [6]. The accuracy of joint estimation by the Kinect skeleton tracking system is comparable to motion capture under controlled conditions of body posture Xu et al. [45], but the validity for general postures or movements has yet to be established [27, 45]. Precise anthropometric measurements may still require direct reference to anatomical labeling of the skeletal system. The requirements for accuracy in sit-to-stand movement using telemetry for risk screening need further investigation in different subject populations.

A second limitation of this study is the potential for bias due to the physical background [10] or sex [16] of the participants on clinical interpretation of the velocity of sit-to-stand motion. In a study of senior athletes, the weight transfer time and center of gravity sway were similar regardless of age or sex, while rising power during sit-to-stand motion decreased with increasing age [10]. This finding suggests that fall risk may be decreased by training, even if the speed of sit-to-stand motion is not improved. The lifespan history of physical training should also be considered in evaluation of sit-to-stand movements. Lastly, in this study, multi-group SEM was conducted in relatively few participants, due to difficulty recruiting male participants. Thus, careful interpretation of the results is required, even though the model evaluation revealed a very good fit to the data.

Practical use of sit-to-stand movement in telemetry will allow periodical evaluation of training outcomes at home to improve muscular strength and screen for the appearance of fall risk. It will be useful to share this information among subjects, physical exercise instructors, and supporters or healthcare personnel for older adults to optimize the protocol of physical intervention and to prevent fall. In this study, both young and older healthy subjects were recruited to evaluate only age-related effects on the evaluation model. All older subjects were active volunteers who were willing to participate in the community club for health promotion, and thus may be a relatively low-risk group for decline in physical activity. A future study is required to evaluate the potential of the method to detect high-risk subjects.

## Conclusion

To develop a telemetry method to monitor physical activity of older adults, performance in sit-to-stand motion in young and older adults was measured using a conventional network camera and relationships with simple physical activity indices were investigated. Findings from multi-group SEM suggested that muscular strength of the lower limbs reflected ability in motion skills, and that IEMV may be a predictor of muscular strength. The results suggest that IEMV computed by image analysis of sit-to-stand motion recorded by a conventional webcam is sufficient to predict muscular strength in older adults. Therefore, remote video monitoring of sit-to-stand motion is a safe and effective approach for older adults who are unwilling to participate in group-exercise programs, and will allow assistance with physical examinations and instructions to support wellness of community-dwelling older adults.

## Abbreviations

2D, two-dimensional; 3D, three-dimensional; AC, arm curl; AIC, Akaike information criterion; AVI, Audio Video Interleave; BCC, Browne-Cudeck Criterion; CFI, comparative fit index; F8W, figure-of-eight walk; FR, functional reach; GFIs, goodness-of-fit indices; HR, heel rise; ICT, information and communication technology; IEMV, iliac elevation maximum velocity; JPEG, joint photographic experts group; KEMAV, knee extension maximum angular velocity; QOL, quality of life; RMSEA, root mean square error of approximation; SEM, structural equation modeling
